# Curcumin Alleviates the Osteogenesis Inhibition and the Aging Process in BMSCs Induced by Iron Overload Through Activating the NRF2/GPX4 Pathway

**DOI:** 10.1002/ptr.70346

**Published:** 2026-04-28

**Authors:** Jingmin Che, Qing Feng, Zhixia Zhao, Jingying Sun, Weihao Ren, Yangmeng Feng, Cuixiang Xu, Xu‐Hui Li

**Affiliations:** ^1^ Shaanxi Provincial Key Laboratory of Infection and Immune Diseases Shaanxi Provincial People's Hospital Xi'an Shaanxi China; ^2^ Shaanxi Engineering Research Center of Cell Immunology Shaanxi Provincial People's Hospital Xi'an Shaanxi China; ^3^ Frontier Institute of Science and Technology Xi'an Jiaotong University Xi'an Shaanxi China; ^4^ School of Life Sciences, Northwestern Polytechnical University Xi'an Shaanxi China; ^5^ College of Forensic Medicine Xi'an Jiaotong University Health Science Center Xi'an Shaanxi China

**Keywords:** BMSCs, bone loss, curcumin, iron overload, NRF2/GPX4 pathway

## Abstract

Dysregulated proliferation and differentiation of bone marrow mesenchymal stem cells (BMSCs) represent a key pathophysiological mechanism in osteoporosis. Recent studies have demonstrated a significant association between ferroptosis and the advancement of osteoporosis, suggesting that targeting ferroptosis could offer novel therapeutic approaches for osteoporosis treatment. Curcumin, a natural antioxidant, has shown therapeutic potential in bone‐related disorders; however, its precise mechanisms for modulating BMSC function—particularly via ferroptosis‐related pathways—remain poorly characterized. This study investigated whether curcumin alleviates iron overload‐induced BMSC dysfunction by targeting ferroptosis, specifically elucidating its molecular mechanisms in promoting osteogenic differentiation and mitigating cellular senescence. Iron‐overloaded BMSC in vitro models and in vivo murine systems were established to model osteoporosis‐related microenvironments. Curcumin was administered to assess its effects on cellular and systemic outcomes, including bone microstructure, mechanical property, differentiation capacity, senescence markers, iron metabolism, and redox homeostasis by using micro‐CT, RNA‐seq, RT‐qPCR, western blot, immunohistochemical, immunofluorescence, and transmission electron microscope (TEM). Furthermore, *Nrf2* siRNA and the Nrf2 inhibitor ML385 were utilized to interrogate curcumin's mechanism of action in iron‐overloaded BMSCs. In vivo, curcumin treatment significantly attenuated iron overload‐induced bone microstructural damage, mechanical property, and elevated Nrf2 and GPX4 expression in BMSCs. In vitro*,* curcumin mitigated iron overload‐induced ferroptosis in BMSCs by upregulating Nrf2 expression, thereby increasing GPX4 levels. This mechanism consequently delayed cellular senescence and promoted osteogenic differentiation. Our findings establish the Nrf2/GPX4 axis as a critical therapeutic target of curcumin for ameliorating iron overload‐induced osteoporosis. This mechanistic insight provides a foundation for developing novel therapeutics against age‐related and postmenopausal osteoporosis.

## Graphical abstract

Under iron overload conditions, curcumin inhibits cellular senescence and promotes osteogenic differentiation by activating the NRF2/GPX4 signaling pathway in bone marrow mesenchymal stem cells (BMSCs), thereby preventing the onset of osteoporosis.

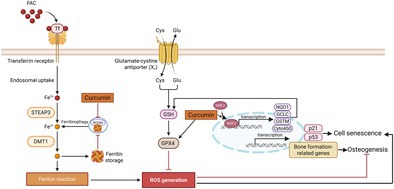

**Abbreviations**
ALP: alkaline phosphatase
ARE: antioxidant response elements
BMSC: bone mesenchymal stem cells
BP: biological process
BSA: bovine serum albumin
BV/TV: Bone volume/Total volume
CC: cellular component
CCK‐8: cell counting kit‐8
Ct.Ar: cortical area
DEGs: differentially expressed genes
DFO: Deferoxamine
ELISA: enzyme‐linked immunosorbent assay
FBS: fetal bovine serum
FDA: Food and Drug Administration
FITC: fluorescein isothiocyanate
FPKM: Fragments Per Kilobase of transcript per Million mapped reads
GPX4: glutathione peroxidase 4
GRAS: Generally Recognized as Safe
GSH: glutathione
Keap1: Kelch‐like ECH‐associated protein 1
KEGG: kyoto encyclopedia of genes and genomes
MF: molecular function
Micro‐CT: micro‐computed tomography
N.Ob/BS: number of osteoblasts/bone surface
NRF2: nuclear factor erythroid 2‐related factor 2
OCN: osteocalcin
PBS: phosphate buffered saline
ROS: reactive oxygen species
SEM: standard error of the mean
Tb.N: trabecular number
Tb.Sp: trabecular separation
Tb.Th: trabecular thickness
TRAP5b: tartrate resistant acid phosphatase 5b
Tt.Ar: total cross‐sectional tissue area
αMEM: modified eagle medium α

## 1 Introduction

Osteoporosis is an asymptomatic skeletal disease that is characterized by reduced bone mass and mineral density, systemic impairment of bone strength, and deterioration of microstructural integrity (Ensrud and Crandall, Ensrud and Crandall 2024). Driven by global population aging, osteoporosis constitutes a major public health burden, affecting over 200 million individuals worldwide (prevalence: 18.3%), with significantly higher rates in women (23.1%) than men (11.7%) (Salari et al. 2021). The pathogenesis involves critical factors such as the senescence of bone marrow mesenchymal stem cells (BMSCs) and the dysregulated adipogenic‐osteogenic balance (Yang et al. 2023; Zeng et al. 2022). While modulating BMSC activity to favor osteogenesis represents a potential therapeutic strategy, effective pharmacological approaches remain limited.

Iron overload is identified as an independent risk factor for the development of osteoporosis (Che et al. 2020; Xia et al. 2023; Yang et al. 2024). Recent investigations indicate significant iron accumulation in individuals with disuse‐induced osteoporosis (Zhang, Zhao, et al. 2021), diabetic osteoporosis (Harrison et al. 2023; Yang et al. 2022; Zhang et al. 2023), and postmenopausal osteoporosis (Zhang, Wang, et al. 2021). Iron accumulation is a prerequisite for ferroptosis, an iron‐dependent form of regulated cell death driven by iron‐mediated lipid peroxidation and reactive oxygen species (ROS) accumulation (Dixon et al. 2012). Evidence indicates that the ferroptosis pathway in BMSCs is activated in the femurs of patients with osteoporosis (Chen et al. 2025; Jing et al. 2023), and the suppression of ferroptosis in BMSCs can reduce cell death (Li, Zeng, et al. 2020). Moreover, targeting ferroptosis in BMSCs can improve the repair of bone abnormalities (Yuan et al. 2024).

Curcumin, a naturally occurring diketone compound derived from rhizomes of Zingiberaceae and Araceae plants, exhibits potent anti‐inflammatory and antibacterial properties, underpinning its clinical use in chronic inflammatory diseases (Priyadarsini 2014; Turer and Sanlier 2025; Xu et al. 2025). Emerging evidence supports its osteoprotective effects, including enhanced bone density (Chen et al. 2021; Li et al. 2024; Yang et al. 2011, 2015).

Nuclear factor erythroid 2‐related factor 2 (NRF2) is an essential target mediating curcumin's activation of cellular antioxidant defenses (Liu, Rokavec, et al. 2023; Rahban et al. 2020) and functions as a key regulatory element in the process of ferroptosis (Dodson et al. 2019; Yan et al. 2023). However, the specific mechanistic link between curcumin‐induced NRF2 activation and ferroptosis mitigation in BMSCs remains poorly understood, particularly regarding its impact on cellular viability and osteogenic differentiation capacity.

To address this knowledge gap, our study integrates complementary in vitro and in vivo models of iron overload, induced via iron metabolism modulators. We comprehensively assessed BMSC survival, osteogenic differentiation markers, and ferroptosis‐related indicators to elucidate the mechanisms by which curcumin modulates BMSC function under iron overload conditions. Our findings establish the Nrf2/GPX4 axis as a critical therapeutic target of curcumin for ameliorating iron overload‐induced osteoporosis. This investigation aims to provide critical insights into the molecular pathways linking phytochemical intervention, iron metabolism, and bone homeostasis, informing future targeted therapies for metabolic bone disorders.

## 2 Material and Methods

### 2.1 Extraction, Culture, and Identification of Original BMSCs

Three‐week‐old male C57BL/6 mice (10–12 g) were obtained from Beijing Vital River Laboratory Animal Technology Co. Ltd. Mice were sacrificed by cervical dislocation. The iliac bones, femurs, and tibiae were harvested, and the soft tissues were excised. The distal extremities of the femora and tibiae, along with the iliac fossa, were removed to expose the medullary cavity. Bones were rinsed with phosphate buffered saline (PBS), and bone marrow was aspirated to generate a single‐cell suspension. Cells were pelleted by centrifugation and washed once with serum‐free alpha‐modified Eagle's medium (αMEM) (PM150421, Procell, Wuhan). Following a second centrifugation, the cell pellet was resuspended in α‐MEM supplemented with 10% fetal bovine serum (FBS) (FSD500, Excellbio, Suzhou). The suspension was plated in cell culture flasks and incubated at 37°C under 5% CO_2_. Cells isolated at this stage were designated passage 0 (P0). Culture medium was replaced the following day. Upon reaching 80% confluence, cells were trypsinized and subcultured, becoming passage 1 (P1). Cells used for experimental procedures were maintained within passages P2 to P6.

### 2.2 Cell Viability Assay

**FIGURE 1:**
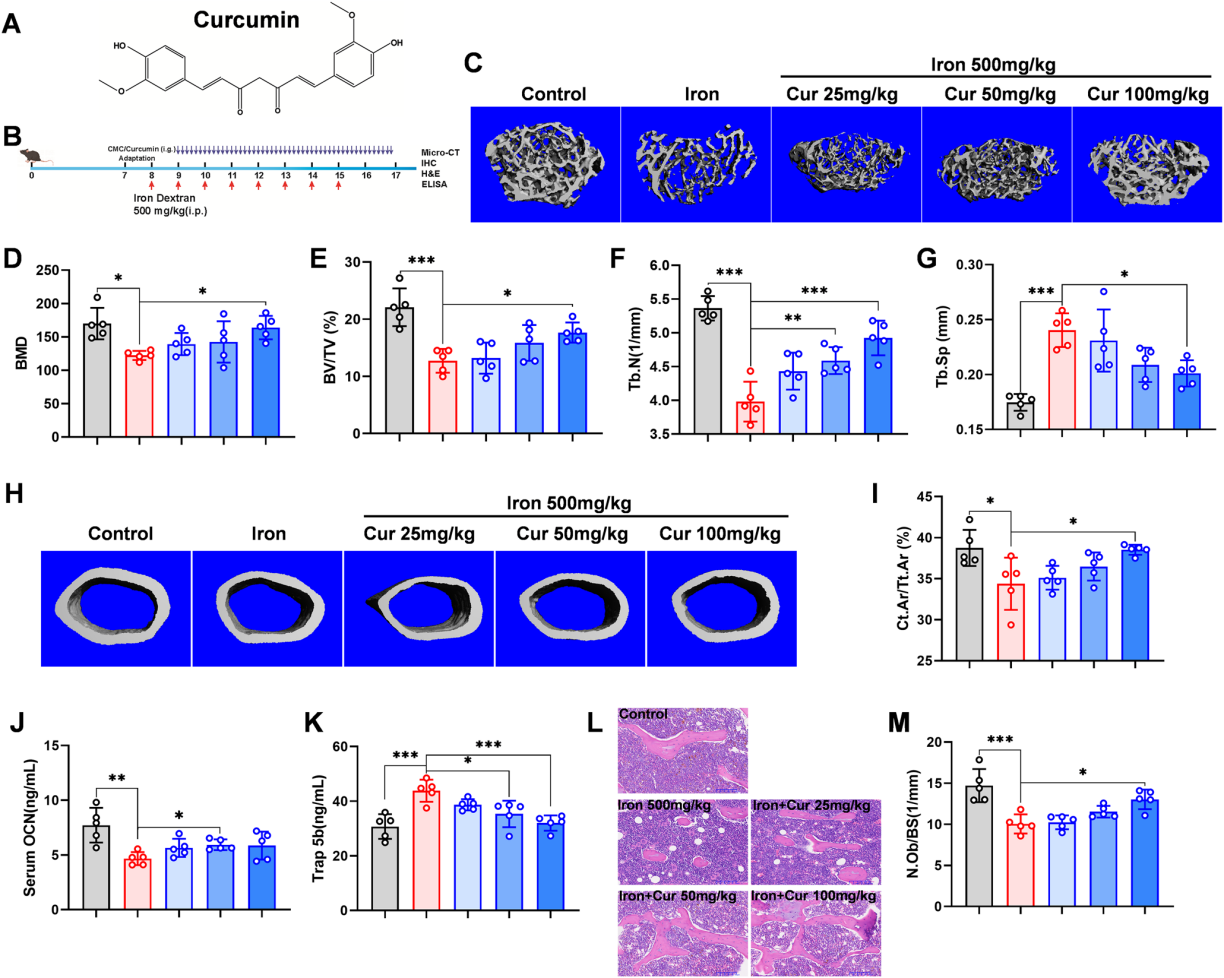
The alleviating effect of curcumin on bone loss induced by iron overload in mice. (A) Schematic diagram of the molecular structure of curcumin; (B) Schematic diagram of the animal experiment design; (C) Three‐dimensional reconstruction image of the trabecular structure of the distal femur; (D) Quantitative analysis of trabecular bone mineral density (BMD); (E) Assessment of trabecular bone volume fraction (BV/TV); (F) Measurement of trabecular number (Tb.N); (G) Analysis of trabecular spacing (Tb.Sp); (H) Three‐dimensional reconstruction image of the cortical structure of the mid‐femur; (I) Assessment of the ratio of cortical area to total area (Ct. Ar/Tt.Ar); (J) Analysis of serum bone metabolism markers (OCN and Trap‐5b) using ELISA kits; (K) H&E staining results of the distal femoral trabeculae (Bar = 100 μm); (L) Evaluation of the number of osteoblasts on the trabecular surface (N.Ob/BS). *n* = 5/group. The values are presented as means ± SD. *p < 0.05, **p < 0.01, ***p < 0.001.

Cells were inoculated at a density of 2.5 × 10^4^ cells/mL, with 200 μL of cell suspension dispensed into each well of a 96‐well plate. Following cell adhesion, culture media with designated curcumin (98.16%, HY‐N0005, MedChemExpress, Shanghai, Figure 1A) concentration was introduced based on cell grouping, and the cells were incubated at 37°C in a 5% CO_2_ environment for 24, 48, and 72 h. Subsequent to the removal of the culture medium, 10% Cell Counting Kit‐8 (CCK‐8) (K1018, APExBio, Houston) in serum‐free medium was introduced to each well and incubated at 37°C for 2 h, after which the optical density values at 450 nm were measured using a microplate reader.

### 2.3 β‐Gal Staining Experiment

Cells were seeded at a density of 2.5 × 10^4^ cells/mL, with 200 μL of cell suspension added to each well of a 96‐well plate. After cell adhesion, culture medium containing specified drug concentrations was added according to cell grouping (Control group, FAC 400 μM group (1185‐57‐5, sigma, USA), FAC 400 μM+ Cur 2.5 μM, FAC 400 μM+ Cur 5 μM, FAC 400 μM+ Cur 10 μM), and the cells were further incubated at 37°C in a 5% CO_2_ incubator for 48 h. The culture medium was removed, the cells were washed once with PBS, and fixed with 4% paraformaldehyde for 15 min. Subsequently, cells were stained according to the instructions of the β‐Gal staining kit (C0602, Beyotime, Shanghai), and observations were made using an inverted microscope.

### 2.4 Osteogenic/Lipogenic Differentiation Detection of BMSCs

Osteogenic Differentiation Detection: Cells in the logarithmic growth phase were seeded at a density of 6 × 10^^4^ cells/well in a 24‐well plate. Once cell confluency reached 95%, the medium was replaced with osteogenic differentiation induction medium containing specified drug concentrations, with medium changes every 2 days. The cells were incubated in a 37°C, 5% CO_2_ incubator for 7 days, after which the medium was removed, and cells were washed once with PBS and fixed with 4% paraformaldehyde for 30 min. Cells were stained using the BCIP/NBT alkaline phosphatase detection kit (C3206, Beyotime, Shanghai) and observed under a microscope for photography.

Lipogenic Differentiation Detection: During the logarithmic growth phase, cells were seeded at a density of 6 × 10^4^ cells/well in a 24‐well plate. Once cell confluency reached 95%, the culture medium was replaced with lipogenic differentiation induction medium containing specified drug concentrations. The medium was changed every 2 days, and the cells were incubated in a 37°C, 5% CO_2_ incubator for 12 days. Subsequently, the medium was removed, and cells were washed once with PBS, fixed with 4% paraformaldehyde for 30 min. Cells were then stained using the Oil Red O staining kit (C0157S, Beyotime, Shanghai) and observed under a microscope for photography.

### 2.5 RNA Extraction and RNA‐Seq Sequencing Analysis

Cells were seeded at a concentration of 0.75 × 10^4^ cells/mL in a six‐well plate. After cell adhesion, the medium was replaced with culture medium containing specified drug concentrations, and the cells were incubated at 37°C in a 5% CO_2_ environment for 48 h. The culture medium was then removed, and cells were washed once with PBS. Total RNA was extracted from treated cells using TRizol reagent (15596018, ThermoFish, USA). After quality control, a total amount of 1 μg RNA per sample was used as input material to establish DNA libraries for transcriptome sequencing by Novogene Technology Co. Ltd. (Tianjin, China). Raw reads were generated and filtered. The clean reads were used for mapping to the reference genome using Hisat2 v2.0.5 software featureCounts v1.5.0‐p3 was used to count the reads numbers mapped to each gene. And then FPKM of each gene was calculated based on the length of the gene and reads count mapped to this gene. Differential expression analysis of two groups (two biological replicates per condition) was performed using the DESeq2 R package (1.20.0). Genes with an adjusted *p*‐value < 0.05 found by DESeq2 were assigned as differentially expressed. Kyoto Encyclopedia of Genes and Genomes (KEGG) pathway enrichment analysis of differentially expressed genes was implemented by the clusterProfiler R package, in which gene length bias was corrected. The expression levels of ferroptosis‐related differentially expressed genes were normalized using FPKM (Fragments Per Kilobase of transcript per Million mapped reads) and visualized through a heatmap. Each tile in the heatmap represents the normalized value, where color intensity reflects expression magnitude: heightened red hues indicate elevated expression levels, while intensified blue tones denote reduced expression levels.

### 2.6 Total Iron and Ferrous Ion Content Detection

Cells were seeded at a concentration of 0.75 × 10^4^ cells/mL in a six‐well plate. After cell adhesion, culture medium containing specified drug concentrations was added according to cell grouping, and cells were incubated for 48 h. The culture medium was then removed, and cells were washed once with PBS, followed by trypsin digestion and centrifugation to collect the cells. An appropriate amount of lysis solution was added to lyse the cells, and the total iron content detection kit's (I291, DOJINDO, Japan) instructions were followed to measure the levels of ferrous and ferric ions, with normalization to protein content.

### 2.7 ROS and Mitochondrial Membrane Potential (MMP) Detection

Cells were seeded at a concentration of 0.75 × 10^4^ cells/mL in a six‐well plate. Once the cells adhered, the corresponding stimulus culture medium was added according to cell grouping, and cells were incubated under appropriate conditions for 24 h. The culture medium was then removed, and cells were washed once with PBS. Each well received 500 μL of α‐MEM medium containing 10 mM DCFH‐DA (S0034S, Beyotime, Shanghai) or JC‐1 working medium (C2003S, Beyotime, Shanghai), respectively, and was incubated at 37°C in the dark for 30 min. Finally, observations were made using an inverted fluorescence microscope.

### 2.8 Transmission Electron Microscopy (TEM)

Cells were seeded at a concentration of 7.5 × 10^4^ cells/mL in a six‐well plate. After cell adhesion, the cells were treated with different culture conditions and incubated for 24 h. The culture medium was removed, and cells were washed once with PBS, followed by fixation with 2% glutaraldehyde. Cells were collected using a cell scraper and fixed with 1% osmium tetroxide for 2 h, followed by washing with PBS. The cells underwent gradient dehydration with acetone (50%, 70%, 80%, 90%), followed by embedding in epoxy resin. Ultra‐thin sections of 70 nm were cut from the embedded blocks, stained with uranyl acetate for 30 min, rinsed three times with distilled water, and then stained with lead citrate for 10 min, followed by three rinses with distilled water. After the sections dried, mitochondrial morphology was observed and photographed using TEM.

### 2.9 GSH, MDA, and ATP Detection

Cells were seeded at a concentration of 0.75 × 10^4^ cells/mL in a six‐well plate. After cell adhesion, the corresponding stimulus culture medium was added according to grouping, and the cells were incubated under appropriate conditions for 48 h. The culture medium was removed, and cells were washed once with PBS, followed by lysis with lysis solution and centrifugation to collect the supernatant. The GSH and MDA levels in the cell supernatant were measured according to the instructions of GSH (BC1175, Solarbio, Beijing) and MDA detection kits (BC0025, Solarbio, Beijing). The ATP content in the cell supernatant was measured according to the instructions of ATP detection kits (S0027, Beyotime, Shanghai). And protein content was quantified using the BCA (PC0020, Solarbio, Beijing) method for normalization in statistical analysis.

### 2.10 Cellular Immune Staining

Cells were seeded into six‐well plates at a concentration of 0.75 × 10^4^ cells/mL. After attachment, the appropriate stimulating culture medium was added according to the grouping, and the cells were incubated in a suitable environment for an additional 48 h. Following the removal of the culture medium, the cells were washed once with PBS and fixed with 4% paraformaldehyde. After fixation, the cells were washed three times with PBS; then, the cells were permeabilized with 0.5% Triton X‐100 for 10 min, followed by blocking with 1% bovine serum albumin (BSA) for 30 min. The corresponding primary antibody was added and incubated overnight at 4°C, followed by three washes with PBS; subsequently, the fluorescein isothiocyanate (FITC)‐conjugated secondary antibody was incubated in the dark for 2 h. Finally, after DAPI staining, the cells were washed three times with PBS and observed under an inverted fluorescence microscope for imaging and recording.

### 2.11 siRNA Transfection

Cells were seeded in 6‐well plates and grown to approximately 70% confluence, followed by incubation with serum‐free medium. The Nrf2 inhibitor (si*NFR2*) and negative control (siNC) were designed and synthesized by IBSBIO (Shanghai, China). The sequence of si*NRF2* is shown in Table 1. Cells were transfected with si*NRF2* and siNC using X‐tremeGENE (Invitrogen). The efficiency of NRF2 inhibition was validated by RT‐PCR and Western blot analysis at 48 h post‐transfection.

**TABLE 1:** The sequence of si*NRF2*.

	Sense (5′‐3′)	Anti‐Sense (5′‐3′)
si*NRF2*#1	GAGUUACAGUGUCUUAAUACG	UAUUAAGACACUGUAACUCGG
si*NRF2*#2	GGAUGAAGAGACCGGAGAAUU	UUCUCCGGUCUCUUCAUCCAG
si*NRF2*#3	GAGGAUGGGAAACCUUACUUU	AGUAAGGUUUCCCAUCCUCUU

### 2.12 RT‐qPCR

Cells were seeded at a density of 7.5 × 10^4^ cells/mL, with 2 mL of cell suspension per well in six‐well plates. After cell attachment, the culture medium was replaced with medium containing the appropriate concentration of the drug, and the cells were further cultured for 48 h. Total RNA was extracted from the cells using TRIzol reagent according to the manufacturer's instructions. cDNA was synthesized from 1 μg of RNA using SuperScript reverse transcriptase (M314C, Promega Corporation). Real‐time quantitative PCR (RT‐qPCR) was performed using SYBR Green PCR Master Mix (4309155, Applied Biosystems). The reaction was conducted on the StepOnePlus real‐time PCR system (Applied Biosystems). The primers used are as follows: *FTH1*: forward: CAAGTGCGCCAGAACTACCA; reverse: GCCACATCATCTCGGTCAAAA; *TFRC*: forward: GATCAAGCCAGATCAGCATTCT; reverse: ACCGGGTGTATGACAATG GTT; *HMOX‐1*: forward: GTCAGGTCCTGAAGAAGATTG, reverse: CATAAATTCCCACTGCCACTG; *NCOA4*:forward: AATTGGGACTGGTAGGATGG, reverse: AATTGGGACTGGTAGGATGG. *Cyto p450*: forward: TGCCGCTTTGAATACAATGACC; reverse: ACCTTGTGTTCAGTTAGCAGC; *NQO1*: forward: AAGGATAGGAAAAGGAGGAGG, reverse: CCTCAGCCATTGTTTGAGCA; *GCLC*: forward: GAACATGAAAGTGGCACAGG, reverse: TCCAGGAAACACGCCTTCC; *GSTM1*: forward: AGAAGTTCAAACTGGGCCTG, reverse: GAGTAGAGCTTCATCTTCTCAG. These target gene mRNA levels were analyzed using the ΔΔCT method, and the relative mRNA fold changes were normalized to Gapdh.

### 2.13 Western Blot

Cells were seeded at a density of 7.5 × 10^4^ cells/mL, with 2 mL of cell suspension per well in six‐well plates. After cell attachment, the culture medium was replaced with medium containing the appropriate concentration of the drug, and the cells were cultured for an additional 48 h. The old medium was discarded, and the cells were washed three times with PBS. Cells were lysed using RIPA buffer containing 1% phosphatase and protease inhibitors, and the supernatant was collected by centrifugation. The supernatant was adjusted to a uniform protein concentration based on cell protein concentrations, then denatured using 5× loading buffer. Proteins were separated by SDS‐PAGE and transferred to PVDF membranes. After blocking with 5% non‐fat milk, the anti‐p21 (28248‐1‐AP, Proteintech, Wuhan), anti‐p53 (10442‐1‐AP, Proteintech, Wuhan), anti‐FTH1 (A19544, Abclone, Wuhan), anti‐TFRC (A5865, Abclone, Wuhan), anti‐NCOA4 (ab314553, Abcam, USA), anti‐HO‐1 (66743‐1‐Ig, Proteintech, Wuhan), anti‐GPX4 (67763‐1‐Ig, Proteintech, Wuhan), anti‐SLC7A11 (32384‐1‐AP, Proteintech, Wuhan), anti‐NRF2 (80593‐1‐RR, Proteintech, Wuhan) and anti‐β‐actin (HRP‐66009, Proteintech, Wuhan) were allowed to bind to the target protein overnight at 4°C, followed by incubation with a specific secondary antibody at room temperature for 2 h. The immunoreactive bands were detected using the ECL (PK10001, Proteintech, Wuhan) method, and quantitative analysis was performed using ImageJ software.

### 2.14 Animal Assay

Seven‐week‐old male C57BL/6 mice (16–18 g) were purchased from Beijing Vital River Laboratory Animal Technology Co. Ltd. All mice were housed in an environment of 24°C ± 2°C with access to clean‐grade pellet feed and sterile water, on a 12‐h light/dark cycle. Bedding, cages, and drinking water were changed every 3 days. Thirty mice were randomly grouped using a randomly‐arranged block randomization method. During the grouping process, we ensured an even distribution of sample sizes in each group to minimize the effects of selection bias and confounding factors. These groups: Control group, iron‐overload group (500 mg/kg/week, i.p., iron‐dextrin (ID), D8517, Sigma, USA), iron‐overload + curcumin 25 mg/kg/d group, iron‐overload + curcumin 50 mg/kg/d group, and iron‐overload + curcumin 100 mg/kg/d group. All mice received intraperitoneal injections of 200 μL of saline solution with or without ID once a week for 8 weeks (Figure 1B). One week after the ID injection, 200 μL of 0.5% carboxymethyl cellulose sodium (HY‐Y1889A, MCE, USA) with different doses of curcumin was administered daily by gavage for 8 weeks, after which all mice were anesthetized by inhalation of isoflurane (1%–3%) for sampling and analysis. The design of the animal experiment for mechanism verification is as follows: 20 mice (seven‐week‐old) were randomly divided into four experimental groups, specifically as follows: Control group, iron‐overload group (500 mg/kg/week, i.p), iron‐dextrin (ID), iron‐overload + curcumin (100 mg/kg/d, i.g) group, iron‐overload + Ferrostatin‐1 (5 mg/kg, i.p twice week) group. All mice were anesthetized by inhalation of isoflurane (1%–3%) for sampling and analysis after 8 weeks. The Animal Care and Use Committee of Xi'an Jiaotong University (IDs: No. 2024–1252) approved all experiment protocols.

### 2.15 Micro‐Computed Tomography (Micro‐CT) Detection

The right femur of each mouse was fixed in 4% paraformaldehyde for 48 h, then transferred to 75% ethanol for fixation, followed by micro‐computed tomography (Micro‐CT, SkyScan 1176; Bruker, Kontich, Belgium). The relevant scanning parameters of the instrument were resolution 9 μm, rotation angle 360°, voltage 80 kV, and current 305 μA. Three‐dimensional reconstruction was performed using NRecon software, and sample analysis was conducted with CTAn software. The distal femur region of interest for trabecular bone analysis was selected from 0.5 to 1.5 mm, with a grayscale threshold set at 76. Three‐dimensional reconstructions were performed on all trabecular slices. Analysis metrics included calculating bone mineral density (BMD, mm^3^), bone volume fraction (bone volume/Total volume, BV/TV, %), trabecular separation (Tb. Sp, mm) and trabecular number (Tb.N, 1/mm). The analysis of the microstructural changes in cortical bone began 5 mm from the growth plate of the distal femur and extended proximally for 1 mm, with a threshold set at 105. The primary analysis metrics included cortical area (Ct. Ar) and total cross‐sectional tissue area (Tt.Ar).

### 2.16 Biomechanical Testing

The biomechanical parameters of the tibias were evaluated by conducting a three‐point bending test utilizing the Intron‐5943 (Norwood, MA, USA), a material mechanics testing equipment. The tibia was loaded at a rate of 1 mm/min until the femur was fractured. The fracture surface of the tibia was approximated as an ellipse. The outer and inner diameters of the long axis and the short axis of the bone fracture section were measured using a Digital Microscope KH8700 (HIROX, Japan). The mechanical characteristics of the tibia and analytical methods refer to those previously reported (Jepsen et al. 2015). The mechanical characteristics parameters of the femur include Toughness (N/mm^2^), Ultimate Load (N), Elastic Modulus (GPa), and Ultimate stress (N/mm^2^).

### 2.17 Biochemical Assay

The blood samples were centrifuged, and the serum was collected. The osteocalcin (OCN) and tartrate‐resistant acid phosphatase 5b (TRAP5b) in serum were measured by using mouse enzyme‐linked immunosorbent assay (ELISA) kits. Mouse OCN ELISA kit (JL19437, JONLNBIO, China) and mouse TRAP5b ELISA kit (JL13353, JONLNBIO, China) were used in these assays, and all steps followed the manufacturer's instructions.

### 2.18 Tissue Immunohistochemical Analysis

The fixed femurs were subjected to decalcification using a decalcifying solution at pH 7.5 at 4°C, with the decalcifying solution replaced daily for a total duration of 24 days. After decalcification, the samples were dehydrated through 10%, 20%, 50%, 70%, and 90% ethanol, followed by clearing in xylene, and finally embedded in paraffin. Sections of 5 μm thickness were cut from the femur. The obtained femur sections were subjected to H&E staining, TRAP, NRF2, and GPX4 staining, with imaging captured using a microscope. Image‐Pro Plus software was utilized to calculate the number of osteoblasts and osteoclasts in both the distal femur trabecular bone and cortical bone, comparing these numbers to the perimeter of the trabecular and cortical bone to determine the number of osteoblasts per bone surface (number of osteoblasts/bone surface, N.Ob/BS), and the positive rates of NRF2 and GPX4 cells in the bone marrow were also calculated.

### 2.19 Statistical Data Analysis

The results were expressed as mean ± SD. Statistical differences between groups were assessed using a one‐way analysis of variance (ANOVA). Two groups were compared using two‐tailed Student's *t*‐test using Graph Pad Prism 8.0 (Graph Pad Software Inc., San Diego, CA). A Bonferroni test was used to correct multiple comparisons. *p*‐values less than 0.05 were considered significant (*p < 0.05, **p < 0.01, ***p < 0.001).

## 3 Results

### 3.1 Curcumin's Inhibitory Effect on Osteoporosis Induced by Iron Overload

Curcumin was fed to iron‐overloaded mice through gavage at different dosages for a duration of 8 weeks for sample assessment (Figure 1B). Micro‐CT analysis indicated that the iron‐overloaded group had markedly reduced trabecular bone mineral density, trabecular bone volume percentage, and trabecular number in the femur relative to the control group, although trabecular porosity was significantly elevated. After curcumin administration, the trabecular bone mineral density, trabecular bone volume percentage, and trabecular number in the femur of iron‐overloaded animals exhibited a dose‐dependent increase, accompanied by a dose‐dependent decrease in trabecular porosity (Figure 1C–G). Analysis of cortical bone characteristics revealed (Figure 1H) that the femoral cortical area fraction was markedly diminished in iron‐overloaded animals relative to controls. In comparison to the iron‐overloaded group, mice administered curcumin demonstrated a dose‐dependent enhancement in femoral cortical area fraction (Figure 1I). Serum biochemical examination revealed that iron‐overloaded mice exhibited markedly reduced levels of the bone formation marker osteocalcin (OCN) relative to controls, which increased in a dose‐dependent manner after curcumin administration (Figure 1J). In contrast, Trap5b levels were markedly elevated in the serum of iron‐overloaded animals relative to controls and diminished in a dose‐dependent fashion following curcumin treatment (Figure 1K). The statistical analysis of osteoblast quantities on the femoral cortical bone surface indicated that iron‐overloaded animals exhibited a considerably reduced number of osteoblasts on the trabecular surface in comparison to the control group. Curcumin therapy markedly elevated the quantity of osteoblasts on the trabecular surface of iron‐overloaded mice in a dose‐dependent fashion (Figure 1L,M). Alterations in the functionality of bone marrow mesenchymal stem cells are a critical mechanism contributing to osteoporosis. Consequently, we then concentrated on examining the regulatory impacts of curcumin on the functionality of bone marrow mesenchymal stem cells in the context of iron overload.

### 3.2 Inhibitory Effects of Curcumin on the Insufficient Vitality and Senescence of BMSCs Induced by Excessive Iron

**FIGURE 2:**
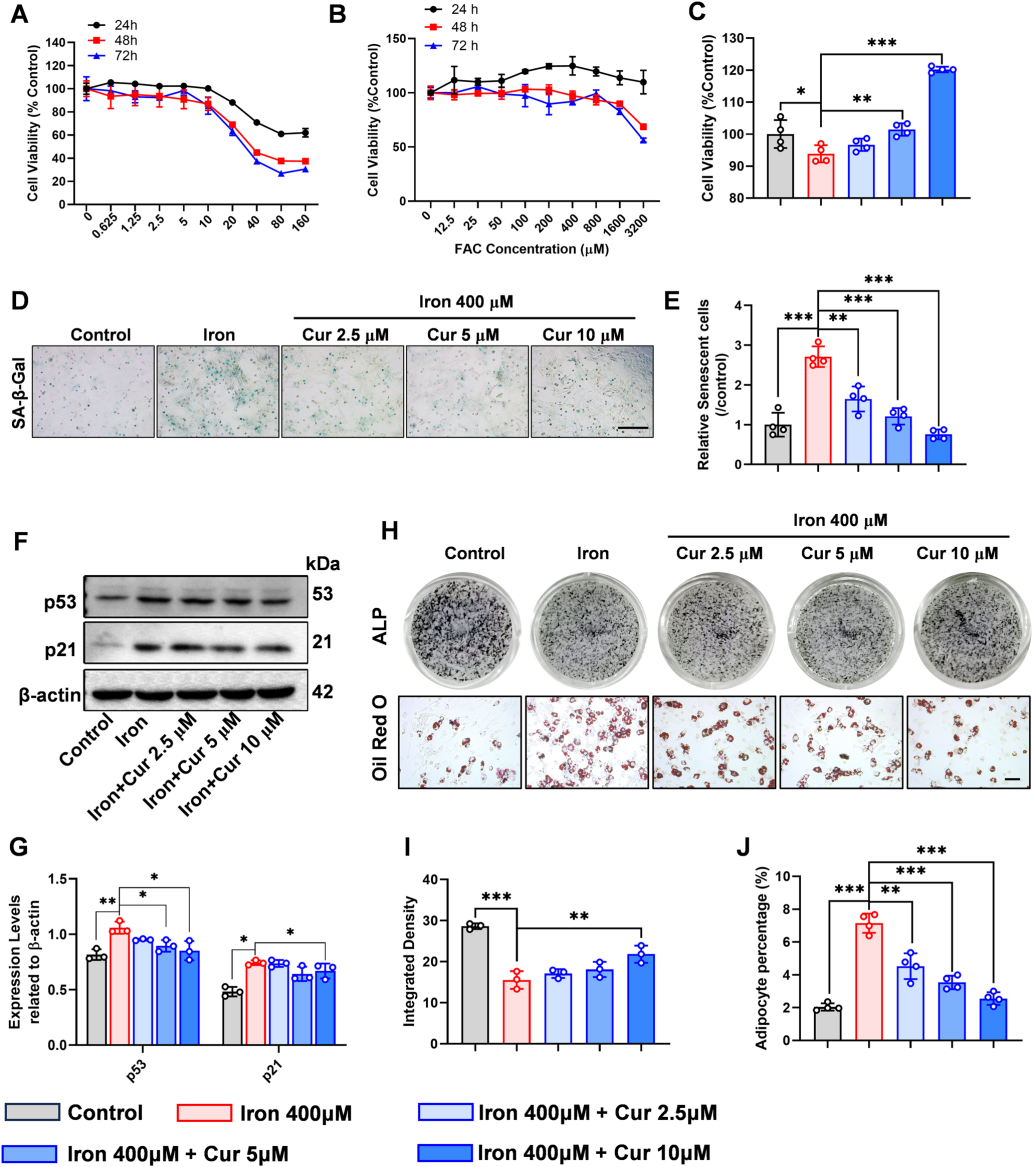
Curcumin inhibits the senescence of BMSCs and promotes their osteogenic differentiation under iron overload conditions. (A) Effect of different concentrations of curcumin on BMSC viability, detected by CCK‐8 assay (*n* = 4/group); (B) Effect of different concentrations of FAC on BMSC viability, detected by CCK‐8 assay (*n* = 4/group); (C) The effect of curcumin on BMSC viability under iron overload conditions (*n* = 4/group); (D) SA‐β‐Gal staining to evaluate the effect of curcumin on BMSC senescence under iron overload conditions (Bar = 100 μm); (E) Statistical analysis of the effect of curcumin on BMSC senescence under iron overload conditions (*n* = 4/group); (F and G) Quantitative and qualitative analysis of the expression of senescence‐associated proteins (*n* = 3/group); (H) Results of ALP staining and Oil Red O staining (Bar = 50 μm); (I) Statistical results of ALP staining (*n* = 4/group); (J) Statistical results of Oil Red O staining (*n* = 4/group). The values are presented as means ± SD. *p < 0.05, **p < 0.01, ***p < 0.001.

To delve deeper into the mechanistic role of curcumin in regulating iron metabolism in BMSCs, we initially evaluated its impact on BMSC cell viability. The CCK‐8 assay was subsequently employed to evaluate the impact of different curcumin doses on cell viability. The findings demonstrated that curcumin doses under 10 μM did not significantly impact cell viability (Figure 2A). Furthermore, we used FAC to construct a model of iron overload cells. The results showed that 400 μM FAC did not affect the viability of bone marrow mesenchymal stem cells (Figure 2B). Accordingly, doses of 2.5, 5, and 10 μM were chosen for further testing. After administering various concentrations of curcumin in conjunction with iron, the findings indicated that curcumin significantly improved cell viability in a concentration‐dependent manner relative to the iron‐only treatment group (Figure 2C). The aging of BMSCs significantly contributes to osteoporosis. SA‐β‐Gal staining was used to evaluate the inhibitory effect of curcumin on BMSC senescence induced by high iron. The findings indicated that, in contrast to the control group, high iron markedly increased BMSC senescence, whereas curcumin successfully mitigated this impact in a concentration‐dependent way (Figure 2D,E). Western blot analysis was performed to assess the expression of senescence‐related proteins, demonstrating that increasing iron levels significantly increased the expression of senescence marker proteins, including p21 and p53. Curcumin, however, mitigated this increase in a concentration‐dependent manner (Figure 2F,G). The disparity between adipogenic and osteogenic development of BMSCs is a significant determinant in osteoporosis. Alkaline phosphatase (ALP) staining results demonstrated that, relative to the control group, elevated iron levels markedly diminished the differentiation of BMSCs into osteoblasts, while curcumin enhanced ALP expression in a dose‐dependent fashion (Figure 2H,I). The results of Oil Red O staining indicated that, relative to the control group, elevated iron levels significantly increased the differentiation of BMSCs into adipocytes, whereas curcumin exhibited a dose‐dependent inhibition of adipocyte formation (Figure 2H,J). Consequently, curcumin may promote the osteogenic differentiation of BMSCs and inhibit BMSCs senescence by inhibiting the increase of cellular iron content.

### 3.3 Ferroptosis May Elucidate the Mechanism via Which Curcumin Influences the Impact of Iron Excess on BMSCs

**FIGURE 3:**
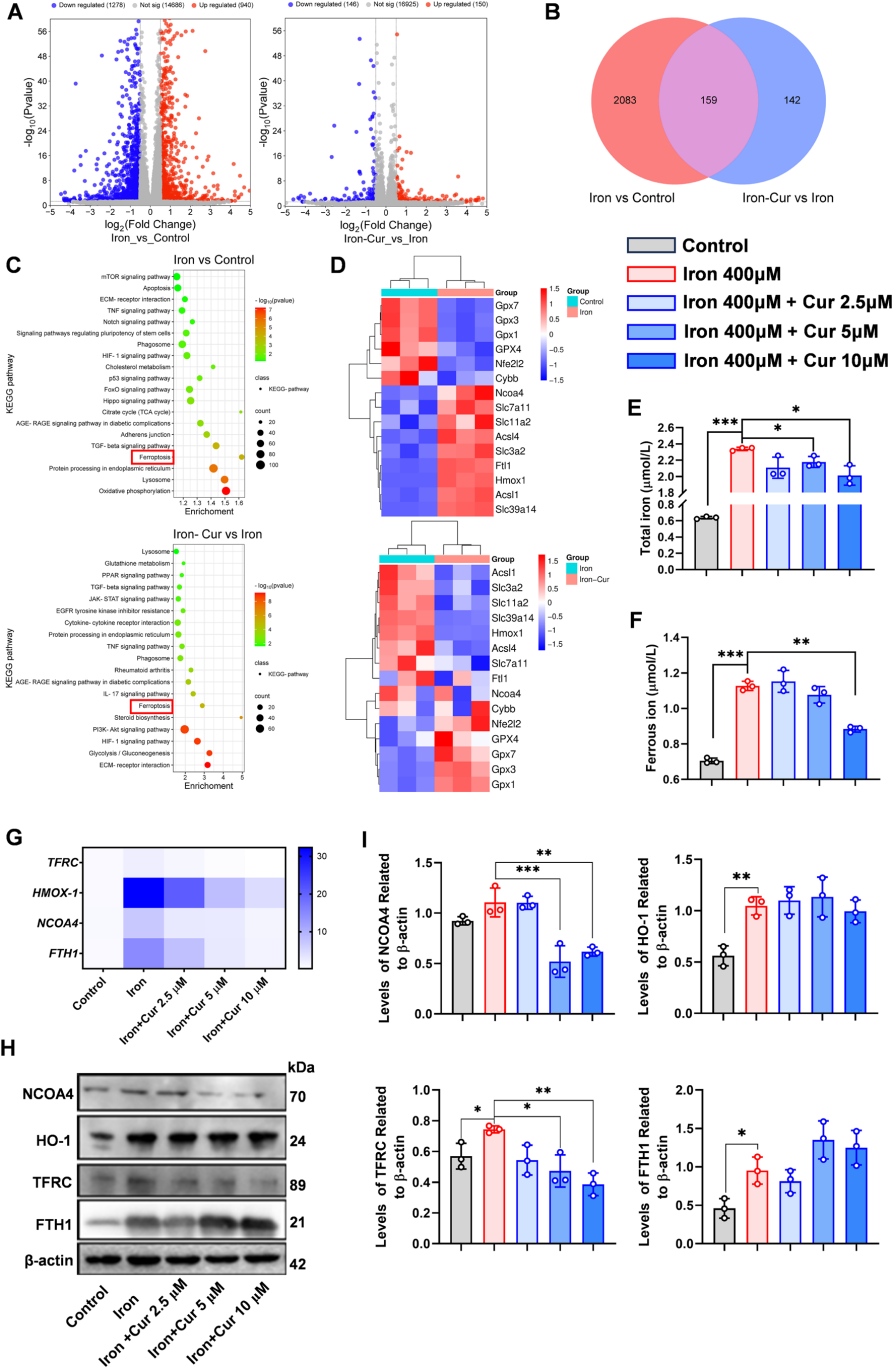
Curcumin regulates iron metabolism levels in BMSCs under iron overload conditions. (A) Volcano plot of differentially expressed genes between the iron overload group, control group, and iron‐curcumin group; (B) Venn diagram of 2384 differentially expressed genes; (C) KEGG pathway enrichment analysis of differentially expressed genes in BMSCs treated with 5 μM curcumin under 400 μM iron treatment; (D) Clustering heat map of iron death‐related gene expression; (E) Measurement results of total iron content in cells; (F) Measurement results of ferrous ion content in cells; (G) RT‐qPCR detection of the expression of iron metabolism‐related genes; (H and I) Expression and statistical analysis results of iron metabolism‐related proteins. *n* = 3/group. The values are presented as means ± SD. *p < 0.05, **p < 0.01, ***p < 0.001.

To elucidate the probable pathways by which curcumin inhibits iron overload‐induced senescence and the differentiation of BMSCs into adipose and bone tissues, we performed RNA sequencing on control, iron overload, and iron+curcumin groups. The findings indicated that iron overload treatment resulted in the overexpression of 940 genes and the downregulation of 1278 genes, whereas the incorporation of curcumin into the iron overload group caused the downregulation of 146 genes and the upregulation of 150 genes (Figure 3A). The Venn diagram of differentially expressed genes across the three groups revealed 159 genes that were concurrently regulated by both iron overload and curcumin (Figure 3B). Subsequent KEGG pathway enrichment analysis of these differentially expressed genes revealed ferroptosis as a frequently regulated signaling pathway (Figure 3C). Cluster analysis of the enhanced ferroptosis‐related genes indicated that curcumin could counteract the alterations in gene expression caused by iron excess (Figure 3D). Considering that elevated intracellular iron levels are essential for the induction of ferroptosis, we quantified total intracellular iron and ferrous ion concentrations. The findings indicated that curcumin mitigated iron overload‐induced elevations in total intracellular iron and ferrous ions in a concentration‐dependent manner (Figure 3E,F). Analysis of ferroptosis‐related gene expression revealed that iron overload markedly elevated the expression of *HOMX1*, *TRFC*, *NCOA4*, and *FTH1*, but curcumin reduced their expression levels in a concentration‐dependent manner (Figure 3G). Evaluation of ferroptosis‐related protein expression indicated that iron overload significantly increased the levels of TFRC, HO‐1, NCOA4, and FTH1, while curcumin concurrently decreased these protein levels in a concentration‐dependent manner (Figure 3H,I). Consequently, ferroptosis may serve as a vital mechanism by which curcumin mitigates iron overload‐induced senescence and the differentiation of BMSCs into adipose and bone tissues.

### 3.4 Curcumin May Modulate Iron Metabolism Through the Activation of the NRF2/GPX4 Signaling Pathway

**FIGURE 4:**
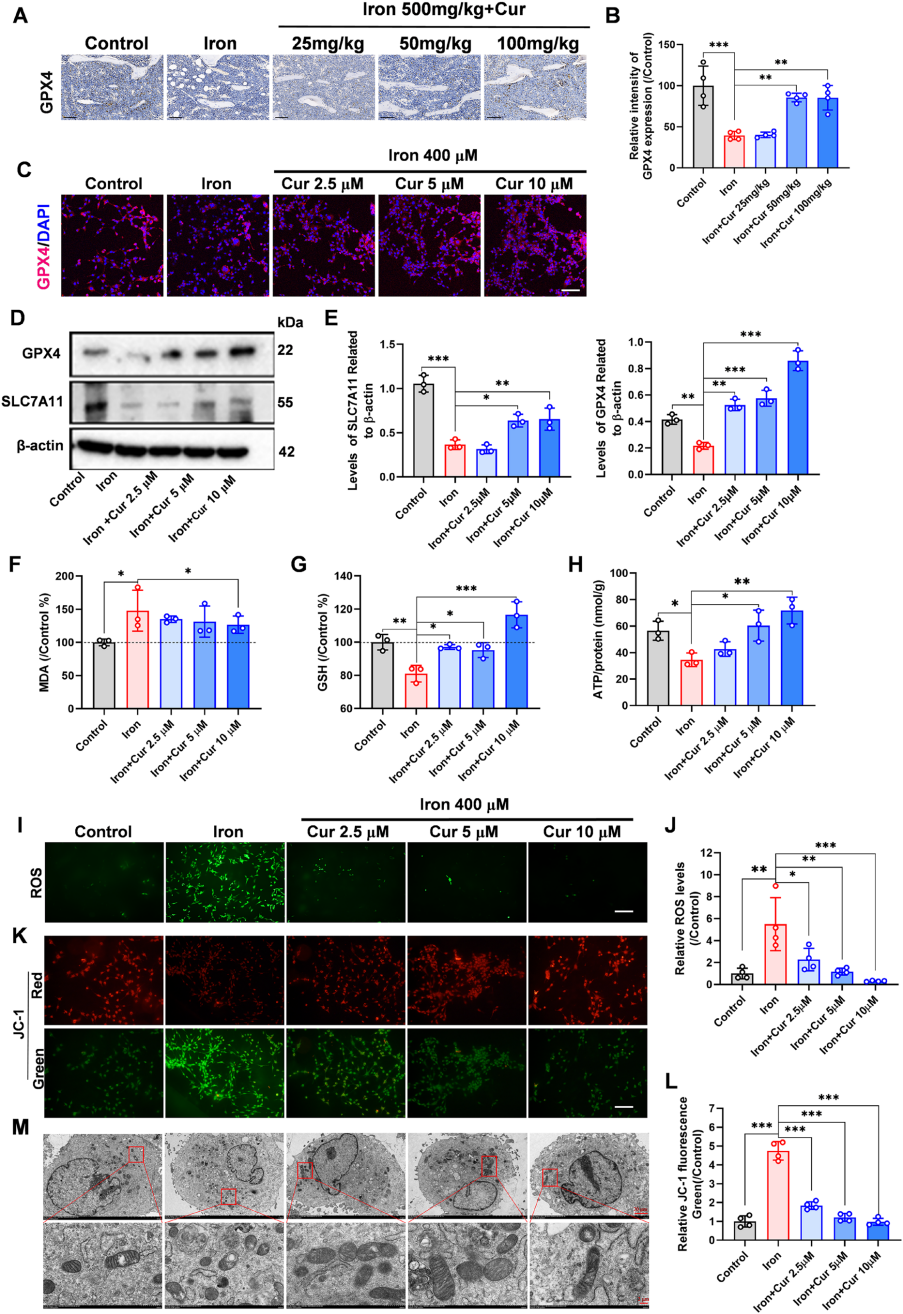
Curcumin inhibits iron overload‐induced iron death in BMSCs. (A) GPX4 immunohistochemical staining results in the femur of iron overload mice (Bar = 100 μm); (B) Statistical analysis of GPX4 expression (*n* = 4/group); (C) GPX4 staining results of BMSCs treated with 400 μM FAC and set concentrations of curcumin (Bar = 100 μm); (D and E) Expression and statistical analysis of iron death‐related proteins (*n* = 3/group); (F) Measurement of malondialdehyde (MDA) content in cells (*n* = 3/group); (G) Measurement of glutathione (GSH) content in cells (*n* = 3/group); (H) Measurement of ATP content in cells (*n* = 3/group); (I and J) Measurement and statistical analysis of reactive oxygen species (ROS) levels in cells (Bar = 50 μm) (*n* = 4/group); (K and L) Measurement and statistical analysis of mitochondrial membrane potential in cells (Bar = 50 μm) (*n* = 4/group); (M) Observation of ferroptosis characteristics under transmission electron microscopy. The values are presented as means ± SD. *p < 0.05, **p < 0.01, ***p < 0.001.

Iron accumulation is a critical factor triggering ferroptosis. The glutathione‐glutathione peroxidase 4 (GSH‐GPX4) antioxidant system is essential in the mechanism of ferroptosis (Tang et al. 2021). Immunohistochemical analysis demonstrated that the expression level of GPX4 in the femoral bone marrow of iron‐overloaded mice was markedly reduced compared to the control group, whereas in iron‐overloaded mice administered curcumin, the expression level of GPX4 was significantly elevated relative to the iron‐overloaded group (Figure 4A,B). The cellular‐level results of GPX4 staining demonstrated that, in comparison to the control group, GPX4 expression was markedly diminished in the excessive iron group, whereas GPX4 expression exhibited a dose‐dependent increase following curcumin administration (Figure 4C). The analysis of ferroptosis‐related proteins indicated that excessive iron ions markedly diminished the expression levels of intracellular GPX4 and SLC7A11 in BMSCs; however, curcumin was able to enhance the expression of these proteins in a concentration‐dependent manner (Figure 4D,E). The buildup of intracellular lipids is a significant component facilitating ferroptosis. Intracellular MDA levels revealed a considerable rise in the excessive iron group compared to the control group, while the addition of curcumin resulted in a dose‐dependent reduction of MDA content (Figure 4F). The findings regarding intracellular reduced glutathione (GSH) levels indicated that iron overload markedly diminished GSH content in BMSCs, whereas curcumin enhanced intracellular GSH levels in a concentration‐dependent manner (Figure 4G). The ATP content detection results revealed that, in comparison to the control group, the excessive iron group exhibited a significant reduction in ATP levels, whereas the addition of curcumin resulted in a dose‐dependent rise in ATP content (Figure 4H). Excessive iron ions can induce the generation of intracellular ROS. The outcomes of fluorescence labeling to assess intracellular ROS concentration indicated that excessive iron exposure in BMSCs resulted in a notable elevation of ROS levels, whereas curcumin was able to diminish intracellular ROS concentrations in a concentration‐dependent manner (Figure 4I,J). Mitochondria are the principal locus of iron ion‐mediated cellular apoptosis. The detection of cell mitochondrial membrane potential revealed that high iron markedly diminished mitochondrial membrane potential, whereas the addition of curcumin greatly enhanced it (Figure 4K,L). Transmission electron microscopy of mitochondrial morphology indicated that high iron ions adversely affected BMSCs, resulting in notable vacuolation and shrinkage of internal mitochondria, which were markedly ameliorated by curcumin treatment (Figure 4M).

**FIGURE 5:**
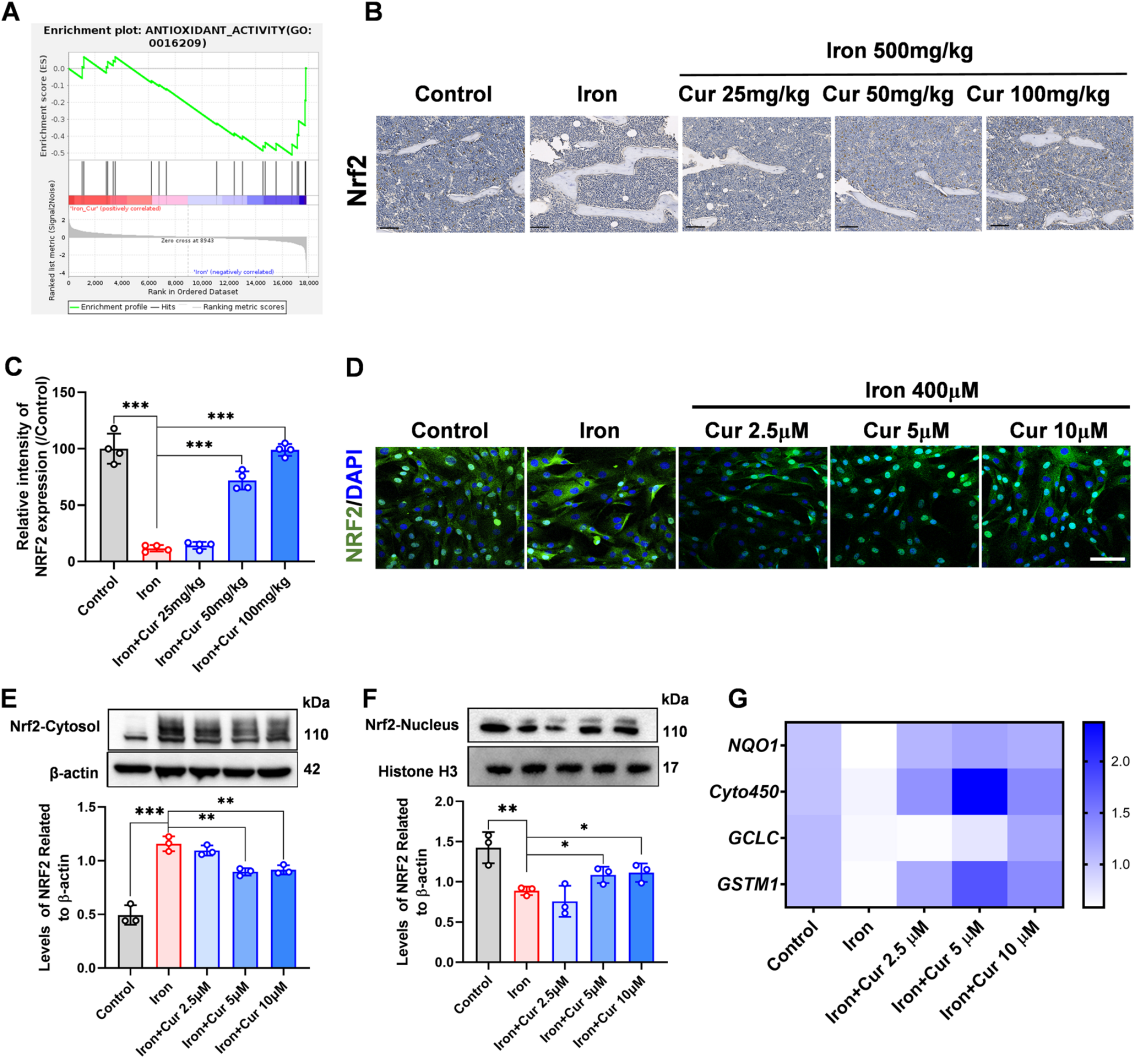
Curcumin promotes the nuclear translocation of NRF2 in BMSCs under iron overload conditions. (A) GSEA enrichment analysis between the iron overload group and the iron control group; (B) Immunohistochemical (IHC) staining of NRF2 in the femur of iron‐overloaded mice (Bar = 100 μm); (C) Statistical analysis of NRF2 expression (*n* = 4/group); (D) NRF staining in BMSCs treated with 400 μM FAC and predetermined concentrations of curcumin (Bar = 50 μm); (E) Expression of NRF2 protein in cytosol and its statistical analysis (*n* = 3/group); (F) Expression of NRF2 protein in nucleus and its statistical analysis (*n* = 3/group). The values are presented as means ± SD. *p < 0.05, **p < 0.01, ***p < 0.001.

Prior research has demonstrated that curcumin improves cellular function by stimulating antioxidant processes within cells. The GSEA analysis results demonstrated that elevated iron levels markedly impeded the activation of the antioxidant system (Figure 5A). NRF2, a pivotal redox transcription factor, significantly contributes to ferroptosis (Yan et al. 2023). Consequently, we employed immunohistochemistry to assess NRF2 expression levels in the femoral bone marrow of mice. The findings indicated that, in contrast to the control group, NRF2 expression in the femoral bone marrow of iron‐overloaded mice was markedly diminished, whereas NRF2 expression levels in the femoral bone marrow of iron‐overloaded mice administered curcumin were significantly elevated compared to the iron‐overloaded group (Figure 5B,C). NRF2 immunofluorescence labeling results at the cellular level demonstrated that high iron suppressed the nuclear transcription of intracellular NRF2, whereas the introduction of curcumin, particularly at a dose of 5 μM, markedly enhanced NRF2 nuclear transcription (Figure 5D). The separation of cell nuclei and cytoplasm, followed by Western blot analysis, revealed that varying doses of curcumin can augment NRF2 nuclear transcription activity in situations of high iron, hence activating the cellular antioxidant system. The cytoplasmic NRF2 content was dramatically diminished alone at a curcumin dose of 5 μM (Figure 5E,F). The findings indicate that curcumin stimulates the antioxidant system through the activation of NRF2 nuclear transcription, consequently preventing the senescence of BMSCs and the decline in osteogenic differentiation induced by excessive iron. These results suggest that curcumin activates the antioxidant system by activating NRF2 nuclear transcription, thereby inhibiting the senescence of BMSCs and the reduction of osteogenic differentiation caused by excessive iron.

### 3.5 si*NRF2* and ML385 Inhibited the Protective Effects of Curcumin Against Ferroptosis and Cellular Senescence

**FIGURE 6:**
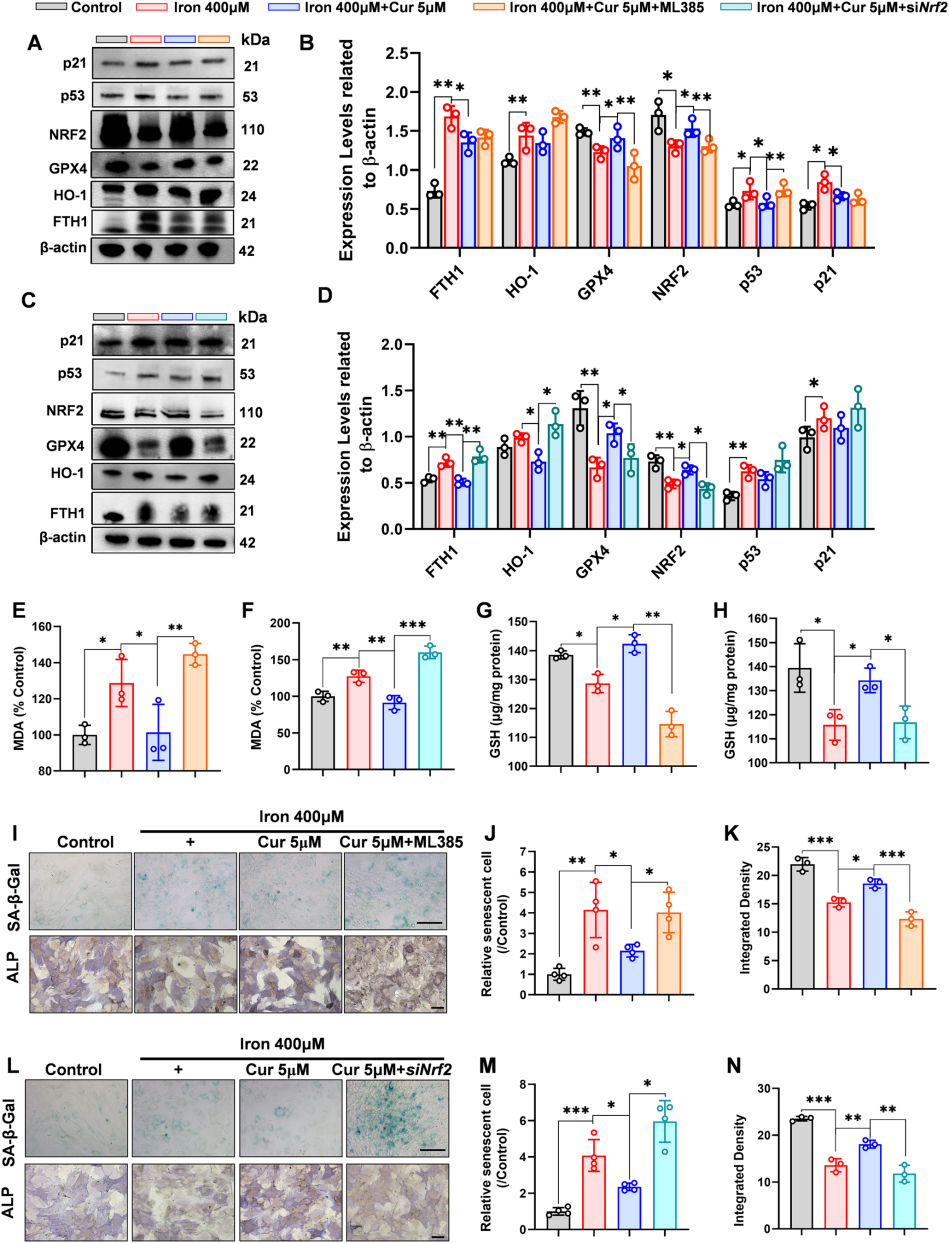
Inhibition of NRF2 significantly reduces the regulatory effects of curcumin on BMSCs under iron overload conditions. (A and B) Effects of ML385‐mediated NRF2 inhibition on the expression of iron metabolism‐related proteins in BMSCs and their statistical analysis; (E) Expression of NRF2 protein in cytosol its statistical analysis (*n* = 3/group); (C and D) Effects of NRF2 knockdown on the expression of iron metabolism‐related proteins in BMSCs and their statistical analysis; (E) Expression of NRF2 protein in cytosol its statistical analysis (*n* = 3/group); (E and F) Detection of intracellular MDA levels (*n* = 3/group); (G and H) Detection of intracellular GSH levels (*n* = 3/group); (I–K) Staining and statistical analysis of β‐Gal positive cells (Bar = 100 μm) and ALP expression (Bar = 50 μm) under with or without ML385 inhibition conditions (Bar = 100 μm); (L–N) Staining and statistical analysis of β‐Gal positive cells (Bar = 100 μm) and ALP expression (Bar = 50 μm) under with or without si*Nrf2* inhibition conditions. The values are presented as means ± SD. *p < 0.05, **p < 0.01, ***p < 0.001.

To corroborate the outcomes of our prior investigations, we utilized small interfering RNA technology and inhibitors to modulate intracellular NRF2 expression, therefore reaffirming the regulatory effect of curcumin on BMSCs under situations of iron overload. We initially checked for the silencing sequence of si*NRF2* using RT‐qPCR and Western blot (Figure S1). Cells were subsequently pretreated with si*NRF2* and the NRF2 inhibitor ML385, followed by the introduction of appropriate doses of iron and curcumin to assess alterations in markers associated with iron‐induced cell death and cellular functioning. Research findings show that the inhibition of NRF2 expression by ML385 considerably diminished the regulatory influence of curcumin on ferroptosis‐related proteins in BMSCs generated by iron overload (Figure 6A,B). Likewise, the suppression of NRF2 expression using si*NRF2* yielded effects analogous to those obtained with ML385 (Figure 6C,D). MDA content analysis indicated that curcumin's inhibitory effect on cellular MDA under elevated iron circumstances was diminished following the inhibition of NRF2 expression using ML385 (Figure 6E) and si*NRF2* (Figure 6F). Moreover, measurements of GSH levels indicated that curcumin's enhancing effect on cellular GSH under conditions of iron overload was diminished following NRF2 suppression (Figure 6G,H). In evaluations of cellular senescence and osteogenic differentiation, the inhibitory impact of curcumin on iron overload‐induced senescence in BMSCs was markedly diminished following the suppression of NRF2 expression via ML385 (Figure 6I,J) and si*NRF2* (Figure 6L,M), especially with si*NRF2*. Furthermore, the inhibitory impact of curcumin on iron overload‐inhibited osteogenic differentiation in BMSCs was markedly diminished following the suppression of NRF2 expression via ML385 (Figure 6I,K) and si*NRF2* (Figure 6L,N). The data indicate that in settings of iron overload, curcumin suppresses senescence and enhances osteogenic differentiation in BMSCs by activating the NRF2 signaling pathway.

### 3.6 Curcumin Mitigates Osteoporosis Through the Inhibition of Ferroptosis

**FIGURE 7:**
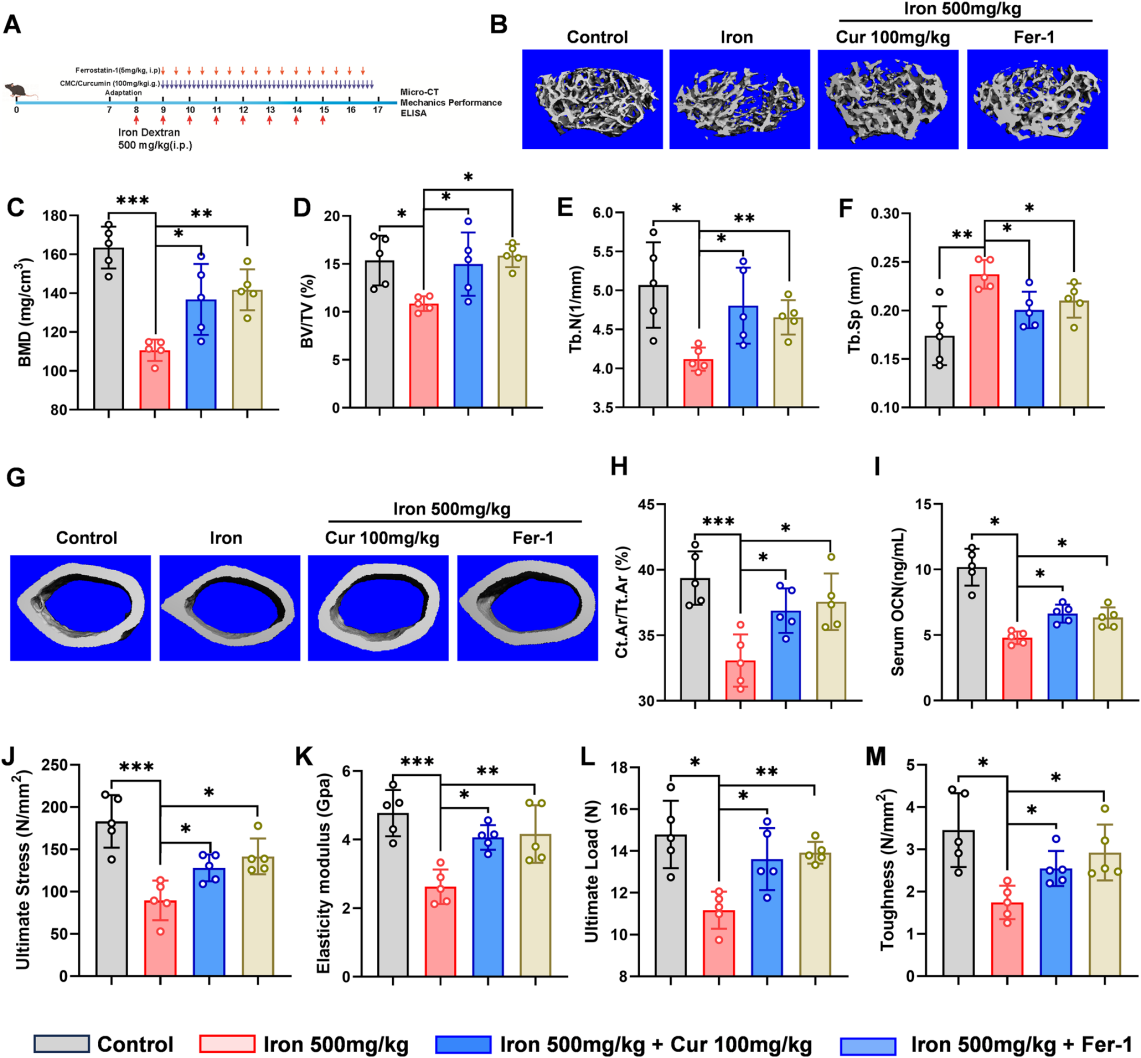
The alleviating effect of curcumin and ferrostatin‐1 (Fer‐1) on bone loss induced by iron overload in mice. (A) Schematic diagram of the animal experiment design; (B) Three‐dimensional reconstruction image of the trabecular structure of the distal femur; (C) Quantitative analysis of trabecular bone mineral density (BMD); (D) Assessment of trabecular bone volume fraction (BV/TV); (E) Measurement of trabecular number (Tb.N); (F) Analysis of trabecular spacing (Tb.Sp); (G) Three‐dimensional reconstruction image of the cortical structure of the mid‐femur; (H) Assessment of the ratio of cortical area to total area (Ct.Ar/Tt.Ar); (I) Analysis of serum bone metabolism markers (OCN) using ELISA kits; Mechanical properties of the tibia in mice were detected through three‐point bending: (J) Ultimate Stress; (K) Elasticity modulus; (L) Ultimate Load; (M) Toughness. *n* = 5/group. The values are presented as means ± SD. *p < 0.05, **p < 0.01, ***p < 0.001.

To determine whether curcumin exerts a protective effect against iron overload‐induced osteoporosis by inhibiting ferroptosis, we conducted a comparative study using the ferroptosis inhibitor Ferrostatin‐1 (Figure 7A). The experimental results indicated that Ferrostatin‐1 significantly inhibited the deterioration of bone microstructure caused by iron overload (Figure 7B–F). Notably, curcumin exhibited comparable inhibitory effects to Ferrostatin‐1, with no statistically significant differences between the two. Similarly, analysis of the cortical bone microstructure demonstrated that Ferrostatin‐1 effectively alleviated the degeneration of cortical bone in mice induced by iron overload (Figure 7G,H), and the protective effect of curcumin in this regard was not significantly different from that of Ferrostatin‐1. Further examination of serum OCN levels revealed that Ferrostatin‐1 significantly promoted bone formation under iron overload conditions (Figure 7I), and the promoting effect of curcumin on bone formation was similar to that of Ferrostatin‐1. Bone mechanical properties are key indicators for evaluating bone quality. Results from three‐point bending tests showed that Ferrostatin‐1 significantly improved the decline in bone mechanical properties caused by iron overload in mice (Figure 7J–M). Importantly, curcumin also significantly enhanced the mechanical properties of bones affected by iron overload, with the extent of improvement being comparable to that of Ferrostatin‐1. In summary, the findings of this study suggest that curcumin effectively alleviates iron overload‐induced bone loss through the inhibition of the ferroptosis pathway.

## 4 Discussion

Bone is a metabolically active tissue whose homeostasis is regulated by a dynamic equilibrium between osteoclast‐mediated bone resorption and osteoblast‐mediated bone formation (Zaidi 2007). During growth and development, bone formation predominates; however, after the age of 30, bone density steadily declines. Traditionally, osteoporosis has been primarily associated with abnormal calcium metabolism. However, recent studies progressively indicate that deregulation of iron metabolism significantly contributes to the development of osteoporosis (Che et al. 2020; Chen et al. 2019; Zhang et al. 2024). Clinical investigations indicate a strong association between increased serum ferritin levels and diminished bone density in postmenopausal women (Cheng et al. 2017). Iron overload has been recognized as an independent risk factor for osteoporosis. Our research further substantiates that excessive iron results in a marked decrease in bone density and trabecular bone volume, while also aggravating the pathological advancement of osteoporosis by diminishing osteoblast populations and bone formation rates.

BMSCs, as multipotent adult stem cells, possess the potential to differentiate into several cell types, including osteoblasts, adipocytes, and chondrocytes, hence playing a crucial role in maintaining bone homeostasis (Yang et al. 2023; Zeng et al. 2022). Osteoblasts are crucial for osseous development through the synthesis and secretion of bone matrix. Research demonstrates that the senescence of BMSCs and the reduction of their osteogenic differentiation potential, leading to diminished bone production rates, are significant factors in the development of senile osteoporosis (Nandy et al. 2024). Our research, alongside that of other scholars, has confirmed that iron overload is not only an independent risk factor for osteoporosis (Che et al. 2020; Mitchell 2012; Zhang et al. 2024) but also a substantial contributor to BMSC aging and reduced osteogenic differentiation capacity. Bone marrow stem cells derived from *HAMP* knockout mice exhibiting iron overload show significantly reduced osteogenic differentiation capacity compared to those from normal mice (Li, Zhang, et al. 2020). This study shows that elevated iron levels hinder the osteogenic development of BMSCs, promote their differentiation into adipocytes, and expedite cellular senescence, hence exacerbating the pathological advancement of osteoporosis.

Ferroptosis is an iron‐dependent, non‐apoptotic type of programmed cell death, characterized by the generation of ROS by the Fenton reaction, leading to lipid peroxidation at the plasma membrane and resulting in ferroptosis (Dixon et al. 2012). In this mechanism, GPX4 suppresses ferroptosis through its antioxidant capabilities (Liu, Wan, et al. 2023; Ursini and Maiorino 2020). Research indicates that iron overload significantly induces ferroptosis and is a crucial element in the differentiation imbalance of BMSCs. Iron overload impedes the development of bone marrow‐derived stem cells into osteoblasts and inhibits the functionality of mature osteoblasts (Balogh et al. 2016). Deferoxamine (DFO), an iron chelator, significantly reduces H_2_O_2_‐induced apoptosis in BMSCs and enhances their resistance to oxidative stress (Khoshlahni et al. 2020). Superparamagnetic iron oxide nanoparticles (ferucarbotran) significantly inhibit the osteogenic differentiation of BMSCs by increasing intracellular iron levels, whereas DFO counteracts the anti‐osteogenic effects of ferucarbotran (Chen et al. 2010). Thus, inhibiting ferroptosis may be an effective approach to mitigate osteoporosis. This study reveals that curcumin promotes the osteogenic differentiation of bone marrow mesenchymal stem cells and inhibits bone loss caused by excess iron, with no significant difference in its inhibitory effect compared to iron death‐specific inhibitors. This indicates that curcumin can effectively suppress iron death in bone marrow mesenchymal stem cells, thereby slowing the progression of osteoporosis.

Curcumin, derived from the rhizomes of *Curcuma longa* L. Initially suggested by Vogel and Pelletier in 1815, it has been traditionally utilized to improve blood circulation, eliminate blood stasis, and alleviate pain (González‐Sarrías et al. 2013). Recent studies have shown that curcumin has several pharmacological properties, such as antioxidant, anticancer, anti‐inflammatory, and antiviral effects, and has displayed considerable therapeutic potential in both preclinical and clinical studies. Furthermore, curcumin is designated as “Generally Recognized as Safe” (GRAS) by the United States Food and Drug Administration (FDA) (Eke‐Okoro et al. 2018). Curcumin exhibits remarkable antioxidant properties, which is why it is widely applied in antioxidant research. Studies have found that a concentration of 10 μM curcumin can alleviate oxidative stress induced by hydrogen peroxide in intestinal cells (Cao et al. 2020). Furthermore, related research indicates that when the concentration of curcumin exceeds 30 μM, it promotes the apoptosis of bone marrow mesenchymal stem cells, whereas concentrations below 20 μM can facilitate their osteogenic differentiation (Chen et al. 2021). In this study, the safe concentrations of curcumin acting on bone marrow mesenchymal stem cells were determined to be 2.5, 5, and 10 μM through the CCK‐8 method, which do not induce cell death and are capable of promoting osteogenic differentiation. Studies demonstrate that curcumin can trigger ferroptosis in clear cell renal cell carcinoma by diminishing NCOA4 levels, thereby hindering the transport of intracellular free iron to lysosomes (Mancias et al. 2014). Nevertheless, the mechanism by which curcumin promotes osteogenic differentiation in BMSCs remains unclear. Our experimental results demonstrate that curcumin inhibits NCOA4 expression in BMSCs. HO‐1 is crucial in regulating ferroptosis, primarily through its increased expression that promotes the breakdown of FTH1, hence raising intracellular free iron levels and initiating ferroptosis (Li et al. 2023). Curcumin has been shown to directly induce HO‐1 expression and enhance FTH1 expression in cells. Our findings indicate that curcumin enhances the expression of HO‐1 and FTH1 in cellular contexts. Curcumin reduces NCOA4 expression during iron overload, suggesting that it inhibits the degradation of FTH1 and the subsequent increase in free iron levels under these conditions.

Nrf2 is an essential regulator of the cellular defense system against oxidative injury. Under normal conditions, Nrf2 associates with Keap1 (Kelch‐like ECH‐associated protein 1), leading to its ubiquitination and subsequent degradation, hence maintaining low basal levels. In the presence of oxidative stress, Nrf2 separates from Keap1, leading to its stability and activation (Suzuki and Yamamoto 2015). Curcumin, a potent antioxidant, inhibits Keap1 activity, hence reducing the ubiquitination and degradation of Nrf2, which enhances Nrf2 stability and facilitates its nuclear translocation. Upon entering the nucleus, Nrf2 binds to antioxidant response elements (ARE), initiating the transcription of downstream antioxidant genes (Jiang et al. 2020; Shahcheraghi et al. 2021). Nrf2 also significantly regulates ferroptosis (Yan et al. 2023). The activation of Nrf2 increases the synthesis of GPX4, an essential negative regulator of ferroptosis, which obstructs lipid peroxidation at the cellular membrane, hence averting ferroptosis (Wen et al. 2023). Nrf2 also regulates the expression of genes related to iron metabolism, such as ferritin heavy chain FTH1 and ferritin light chain FTL, therefore maintaining intracellular iron homeostasis and preventing oxidative damage and ferroptosis due to iron overload (Kasai et al. 2018). In vivo experiments demonstrated that curcumin increases the expression of GPX4 and Nrf2 in bone marrow cells. In vitro assays show that under iron overload conditions, curcumin facilitates Nrf2 nuclear translocation and enhances GPX4 expression in BMSCs, consequently reducing intracellular ROS generation and mitochondrial membrane potential depolarization, ultimately averting ferroptosis. Curcumin primarily reduces cellular aging and promotes osteogenic differentiation in this context.

## 5 Conclusion

In summary, curcumin promotes the osteogenic differentiation of bone marrow mesenchymal stem cells (BMSCs) and delays cellular senescence by inhibiting ferroptosis, thereby effectively mitigating bone loss in mice induced by iron overload. This finding provides an important scientific basis for drug development aimed at osteoporosis in the elderly or postmenopausal women. However, the clinical translation of curcumin still faces several challenges: (1) further research is needed to investigate its therapeutic effects on different types of osteoporosis; (2) the issue of bioavailability when administered directly needs to be addressed; (3) the safety and potential side effects of long‐term use must be evaluated. Addressing these issues will lay the groundwork for the further development of curcumin as a candidate drug for the treatment of osteoporosis.

## Author Contributions

**Jingmin Che:** writing – review and editing, writing – original draft, funding acquisition, project administration, conceptualization, investigation, software. **Qing Feng:** methodology, data curation, visualization, formal analysis. **Zhixia Zhao:** methodology, data curation, formal analysis, validation. **Jingying Sun:** methodology. **Weihao Ren:** methodology, visualization. **Yangmeng Feng:** visualization. **Cuixiang Xu:** project administration, funding acquisition, supervision. **Xu‐Hui Li:** conceptualization, writing – original draft, funding acquisition, writing – review and editing, project administration.

## Funding

This work was supported by the National Natural Science Foundation of China (82401843), the Natural Science Basic Research Program of Shaanxi Province (2024JC‐YBQN‐0846), the Shaanxi Province Key Research and Development Program General Project (2024SF‐YBXM‐046), the Shaanxi innovation ability support plan (2024RS‐CXTD‐84), the Shaanxi provincial health high‐level talent cultivation program, and the Technology Talent Support Program of Shaanxi Provincial People's Hospital (2022JY‐60).

## Conflicts of Interest

The authors declare no conflicts of interest.

## Supporting information


**Figure S1:** The inhibitory effect of siNRF2 on NRF2 expression in BMSCs was verified by RT‐aPCR and WB.

## Data Availability

The data that support the findings of this study are available from the corresponding author upon reasonable request.
