# Supplementary material for: Curcumin Alleviates the Osteogenesis Inhibition and the Aging Process in BMSCs Induced by Iron Overload Through Activating the NRF2/GPX4 Pathway
Source: Phytother Res. 2026 Apr 28;40(7):4188–205. doi: 10.1002/ptr.70346 (PMC13340960; doi:10.1002/ptr.70346)
Supplement: Supplementary file 1 — Figure S1: The inhibitory effect of siNRF2 on NRF2 expression in BMSCs was verified by RT‐aPCR and WB. [file PTR-40-4188-s001.docx]

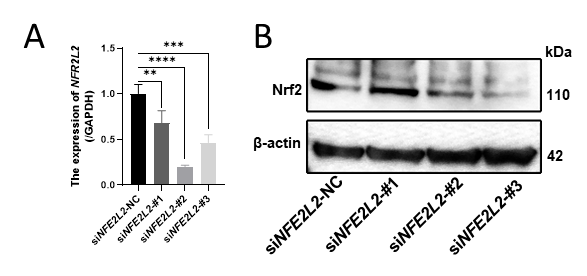


Supplement figure1. The inhibitory effect of siNRF2 on NRF2 expression in BMSCs was verified by RT-aPCR and WB.
